# German version of the specific phobia of vomiting inventory (SPOVI): psychometric properties and correlates in a clinical and non-clinical sample

**DOI:** 10.1186/s12888-025-06744-0

**Published:** 2025-03-31

**Authors:** Severin Hennemann, Antonia Weirich, Adrian Meule, Anne-Kathrin Bräscher, Michael Witthöft

**Affiliations:** 1 Department of Clinical Psychology, Psychotherapy and Experimental Psychopathology, Johannes Gutenberg-University Mainz, Wallstraße 3, 55122 Mainz, Germany; 2 Department of Psychology, University of Regensburg, Universitätsstraße 31, 93053 Regensburg, Germany

**Keywords:** Emetophobia, Fear of vomiting, Specific phobia, SPOVI, German translation

## Abstract

**Background:**

The Specific Phobia of Vomiting Inventory (SPOVI) is a 14-item instrument measuring behavioral avoidance and threat monitoring associated with emetophobia. The current study aimed to provide a German translation of the SPOVI and examine its psychometric properties and correlates.

**Methods:**

*N* = 441 adults from the general population and *N* = 465 outpatients with mental disorders completed the SPOVI and other self-report measures. Factor structure, reliability, convergent, and discriminant validity, as well as clinical utility, were investigated.

**Results:**

The German version of the SPOVI had a one-factor structure and high internal consistency in both samples (ωs > 0.90). Large correlations (*rs* > 0.50) with questionnaires assessing phobic anxiety, general anxiety, illness-related anxiety, and disgust sensitivity supported convergent validity, while relatively low correlations with questionnaires assessing depression, eating disorders or paranoid ideation supported discriminant validity. Among outpatients, those with a specific phobia diagnosis had the highest SPOVI scores.

**Conclusion:**

The German translation of the SPOVI has sound psychometric properties and is a potentially practical clinical screening instrument for emetophobia.

**Pre-registration:**

https://aspredicted.org/5y6zb.pdf.

**Supplementary information:**

The online version contains supplementary material available at 10.1186/s12888-025-06744-0.

## Background

Emetophobia is a specific phobia characterized by an intense fear that oneself or others might vomit. Core clinical features of emetophobia include extensive avoidance behavior (e.g., avoiding individuals likely to vomit, avoiding of social or public situations with a perceived risk of vomiting, or restrictive eating), safety-seeking behavior (e.g., reassurance seeking, cautious food processing, carrying plastic bag or ingestion of antinausea medication), increased sensitivity to bodily sensations (particularly gastrointestinal state and nausea) as well as maladaptive cognition (e.g., “flash-forwards” of imagined vomiting) [1–4]. Vulnerability factors of emetophobia encompass anxiety sensitivity, a predisposition to somatizing anxiety symptoms (particularly gastrointestinal symptoms), disgust propensity (i.e., tendency to frequently experience disgust), and disgust sensitivity (i.e., tendency to perceive disgust as a strongly negative experience), as well as adverse (traumatizing) events such as aversive episodes of vomiting [2, 5–7]. The central appraisal in cognitive-behavioral models is that internal (e.g., gastric symptom perception, vomiting-related thoughts) or external triggers (e.g., situations, persons) lead to hypervigilance towards and misinterpretation of interoceptive sensations as indicators of an imminent vomiting episode, which increases anxiety and arousal, becoming a positive feedback loop for gastrointestinal symptoms and fear of vomiting. According to these models, the symptoms are then maintained by a vicious circle typical of phobic disorders (i.e., avoidance, safety-seeking behavior, hypervigilance, and activation of adverse autobiographical memories) [2, 6, 8].

Intense fear of vomiting is often associated with comorbid mental health issues such as anxiety disorders or obsessive-compulsive disorder (OCD) [4, 9], and can lead to significant functional impairment and reduced quality of life [10, 11]. Emetophobia typically has an onset in adolescence and a lasting persistence with an average illness duration ranging from 12 to 26 years [5].

Although most persons experience disgust or even fear when witnessing vomiting or needing to vomit themselves, only a minority fulfill the criteria for emetophobia. Studies of university students that used the Specific Phobia of Vomiting Inventory (SPOVI) [12] to assess self-reported clinically relevant symptoms of emetophobia based on a proposed cut-off value yielded point prevalences from 4.8% [10] to 9.1% [13]. In a structured clinical interview using the Diagnostic Interview for Mental Disorders [14] in 2064 women from the German general population, a lifetime prevalence of 0.2% as well as a point prevalence of 0.1% of emetophobia (i.e., diagnosed as a specific phobia) was found [15]. Women have reported emetophobic symptoms more often than men with a ratio of 4:1 [4] or 3:1 [13]. Studies on the prevalence of emetophobia in clinical as well as adolescent populations are rare and mostly limited to small samples [12, 16].In contrast, in a survey by Vandereycken [17] that focused on familiarity with and popularity of various mental health problems, almost half (48.6%) of the 111 clinicians (psychologists, physicians, nurses, and social workers) reported having seen cases of emetophobia in their everyday practice.

Yet, emetophobia is often regarded as an overlooked disorder for several reasons. Firstly, its symptoms have a significant overlap with other mental disorders and may thus be more difficult to recognize. In the above-mentioned survey by Vandereycken [17], this was shared among approximately one-third of the clinicians, who indicated that emetophobia is a “variant of another disorder” (p. 149). Examples of phenomenological overlap are hypochondriasis (e.g., fear of becoming sick), panic disorder (e.g., fear of suddenly becoming sick in public), social phobia (e.g., being sick in public and being judged as disgusting), OCD (e.g., compulsive washing, repetitive checking behavior regarding signs of illness in oneself or others), or eating disorders (e.g., food avoidance) [2, 5, 18–20].

Secondly, there is a lack of research on emetophobia when compared to other phobias [5], with a noticeable decline in publications over the past decade that hinders progress in diagnosis and treatment. Treatment guidelines recommend exposure based cognitive behavioral therapy (CBT) for specific phobias in general [21, 22]. CBT approaches for emetophobia often focus on reducing avoidance of vomiting or associated stimuli through exposure (e.g., vomiting themselves, fake vomit, experiencing others vomiting, interoceptive exposure) or behavioral experiments to test expectations (e.g., eating restricted foods or foods that passed the best-before date). However, patients’ willingness to exposure treatment can be limited [9, 23] and clinical anecdotes are propelling the label of patients as being “difficult to treat” [23]. The existing literature on psychological treatments for emetophobia consists mostly of case studies [5]. Examples include acceptance and commitment therapy [24], meta-cognitive therapy [18], exposure-based treatments [23], or other CBT rationales [25, 26] that have been applied to children, adolescents, or adults. High-quality treatment studies are scarce and to the best of our knowledge, only one pilot randomized controlled trial can be found that showed large-sized effects of a 12-session CBT against a waitlist control group, yet in a small adult emetophobic sample only [27].

Currently, there are two validated English self-report measures available for assessing symptoms of emetophobia: the SPOVI [12] and the Emetophobia Questionnaire (EmetQ-13) [16]. Both instruments were developed to provide a brief assessment of the severity of characteristic symptoms of emetophobia – avoidance behaviors and cognitive processes. The scales were designed for diagnostic screening, treatment planning (i.e., identifying maintaining factors to target), and monitoring symptom change. The 14-item SPOVI aims to assess the frequency of avoidance behavior (e.g., situations, persons, images) and threat monitoring (e.g., worrying about vomiting, mental planning) related to the fear of vomiting across the past week. The 13-item EmetQ-13 assesses similar avoidance behaviors, further differentiating between situations/movement/travel, and includes items on expectations about exposure to vomit stimuli. Both instruments may be used concurrently and have demonstrated moderate to large associations in clinical samples [12, 16]. However, the findings on the dimensionality of the SPOVI are inconsistent. The original validation study was performed in the UK with both a clinical sample (*n* = 95 adults diagnosed with emetophobia) and a non-clinical control group (*n* = 90 community sample) and indicated a two-factor structure of the SPOVI with the subscales “avoidance behavior” (e.g., “I have been avoiding adults or children because of my fear of vomiting”) and “threat monitoring” (e.g., “I have been focused on whether I feel ill and could vomit rather than on my surroundings”) [12]. This factor structure was recently corroborated by a Japanese version of the SPOVI in a sample of undergraduate students [28]. On the contrary, a study in a large US-American university student sample provided support for a one-factor model, particularly by demonstrating that in the opposing two-factor model, the two factors correlate almost perfectly, thus possibly not representing independent constructs [29]. Furthermore, the SPOVI and EmetQ-13 have not yet been validated in other languages than English and most recently, Japanese.

## Aims and hypotheses

As the SPOVI has been frequently included in previous studies [5] but evidence for its dimensionality is mixed, this study aimed to provide a German version of the SPOVI and examine its psychometric properties and correlates in two samples, that is a non-clinical mixed community sample and a clinical sample. First, we tested the two-factor model of the SPOVI proposed by the original scale authors Veale et al. [12] and corroborated in the Japanese version [28] against the one-factor model proposed by Maack et al. [29]. Second, we aimed to assess the convergent and discriminant validity of the German SPOVI informed by theoretical conceptualizations of emetophobia [2], empirical data on its associations with other psychopathology [5], and in line with the original development of the SPOVI [12]. We assumed moderate to large positive associations of the SPOVI scores with other measures of phobic anxiety (i.e., intense, excessive, irrational fear of a specific object, situation, or activity that leads to avoidance behaviors and significant distress), including the EmetQ-13 [12] as a concurrent measure of emetophobia. Based on previous evidence [5, 12, 29], we also expected moderately positive associations with other forms of anxiety, that is, general anxiety, social anxiety, and illness-related anxiety as well as with OCD symptoms. We also expected moderately positive associations with the sensitivity to disgust as a central vulnerability factor for emetophobia [7, 30] and with somatosensory amplification, that is, the tendency to experience somatic and visceral sensations as unusually intense, noxious, and disturbing [31], as it is similar to mechanisms proposed in maintaining the vicious circle of emetophobia [2, 6]. Regarding discriminant validity, we expected relatively smaller associations with measures of potentially more unrelated forms of psychopathology such as depression, and eating disorders, in which restricted eating may result from a desire to lose weight (i.e., anorexia nervosa) rather than from fear of vomiting, as well as psychoticism and paranoid ideation. Finally, we exploratively investigated differences in SPOVI scores across various diagnoses of psychotherapy outpatients.

## Methods

### Study design and participants

This study was part of the project “Diagnostics and Intervention for Emetophobia (DIADEM)” and was pre-registered at https://aspredicted.org/5y6zb.pdf.

First, a mixed-community sample was recruited and received an anonymous self-report online survey between December 2022 and February 2023 via the German survey platform https://soscisurvey.de. Participants were recruited via various university e-mail distribution lists (e.g., of the Institute of Psychology of the University of Mainz), social media (e.g., Facebook groups “Emetophobie” and “Emetophobie – Angst vor Übelkeit und Erbrechen“ [Emetophobia - fear of nausea and vomiting]), and the survey was also forwarded to patients at a local migraine and headache clinic. After informed consent was obtained, participants completed all measures, including the SPOVI, via the online survey system. Inclusion criteria were (a) age ≥ 18 years and (b) sufficient knowledge and understanding of the German language. We excluded datasets with missing values (listwise deletion) and incorrect answers on an instructed-response item (“Please select the response category ‘strongly agree’ to show that you have read this sentence.“). Furthermore, we excluded participants with excessive completion time of the survey as an indicator for low quality or even meaningless data using a relative speed index (RSI) value > 2, as recommended by Leiner [32]. The RSI is automatically calculated within the survey platform as the median page completion time (across participants) divided by the individual completion time. A factor of 2 means that the respondent has completed a page twice as fast as the typical respondent. Participants who completed the survey could take part in a voucher raffle (10 × 15 EUR) and psychology students received extra course credit for participation.

Second, a clinical sample was recruited that received a paper-pencil survey, including the SPOVI, as part of the routine diagnostic assessment in the psychotherapy outpatient clinic of the University of Mainz before the initial consultation. The survey was carried out between January 2023 and March 2024. No study-specific inclusion criteria were defined, yet typically only adult outpatients (or minors who are turning 18 before treatment begins) were invited to the routine evaluation and informed consent was obtained. Data were anonymized by the outpatient clinic before analysis. Only complete datasets were included for analyses. The participants received no reimbursement.

### Measures

Demographic characteristics included gender, age, height/weight, relationship status, occupation, and level of education. Contrary to the pre-registration, the Work & Social Adjustment Scale [33] was not included in the survey. In the outpatient sample, datasets also included diagnoses of mental disorders according to DSM-IV, obtained at the beginning of the psychotherapy through the Structured Clinical Interview for DSM-IV (SCID) [34]. Until not otherwise noted, the following questionnaires were used in the mixed community sample.

#### Specific phobia of vomiting inventory (SPOVI)

The SPOVI [12] is a self-report measure consisting of 14 items that assess cognitive and behavioral avoidance behavior during the last week associated with fear of vomiting. These items are answered on a five-point scale from “not at all” (0) to “all the time” (4). Thus, sum scores can range from 0 to 56, with a higher score indicating a greater intensity of emetophobic symptoms. A sum score > 10 has been suggested as a cut-off for indicating the presence of emetophobia [12]. The SPOVI total score demonstrated high internal consistency in clinical (α = 0.91) and non-clinical samples (α = 0.81 – 0.89) [12, 29] and good test-retest reliability for a one-week period [12].

The items of the English version of the SPOVI were forward- and back-translated based on the recommended procedures by Sousa et al. [35] involving independent, native-speaking professional translators as well as consulting with the original author. In a second revision before assessment in the outpatient sample, the response scale of the SPOVI was adapted, as the distance of verbal anchors appeared problematic, given that the minimum and maximum (“not at all” vs. “all the time”) could be either interpreted as indicators of agreement or frequency. Furthermore, the German translation of the words “often” and “a lot” are hardly distinguishable. Therefore, the response categories in the clinical sample were replaced by (0) “never”, (1) “rarely”, (2) “sometimes”, (3) “often”, and (4) “always”. The German version of the SPOVI can be found in Supplement A.

#### Emetophobia questionnaire (EmetQ-13)

The EmetQ-13 [16] includes 13 Items on behavioral avoidance and the dangerousness of exposure to vomit stimuli, which are rated for a one-week time period on a five-point response scale from “strongly disagree” (1) to “strongly agree” (5). Thus, sum scores can range from 13 to 65. Boschen et al. [16] identified a three-factorial structure with the subscales (F1) avoidance symptoms regarding travel, movement, or locations, (F2) perceived danger of exposure to vomit, and (F3) avoidance of others who might vomit. Internal consistency for the total scale was α = 0.82 in emetophobic adults and α = 0.85 in a non-clinical sample [16].

#### Patient health questionnaire-4 (PHQ-4)

The PHQ-4 [36] is a screening instrument consisting of 4 items assessing core symptoms of general anxiety disorder (GAD-2) and depression (PHQ-2) in the past two weeks with two items each. Items are scored on a four-point scale from “not at all” (0) to “nearly every day” (3), and scale scores can range from 0 to 6. The scales demonstrated acceptable internal consistency (PHQ-2: α =.78; GAD-2: α =.75) in a representative sample of the German general population [37].

#### Beck depression inventory-II (BDI-II)

The BDI-II [38] was administered in the clinical sample and includes 21 items covering DSM-IV diagnostic criteria for major depression. Items are answered based on a two-week time period with a list of four individual statements in ascending order of severity or increasing impairment from 0 to 3. Sum scores can range from 0 to 63. The German version of the BDI-II demonstrated high internal consistencies in adults with and without depression (αs > 0.90) [39].

#### Mini – social phobia inventory (MiniSPIN)

The MiniSPIN [40] is a self-report inventory assessing symptoms of social phobia. The inventory consists of three items, which are scored on a five-point scale from “not at all” (0) to “extremely” (4). The items refer to a time frame of one week. The total sum score ranges from 0 to 12. The German version of the MiniSPIN showed good reliability in a clinical (α = 0.83) and representative general sample (α = 0.80) [40].

#### Modified short version of the health anxiety inventory (MK-HAI)

The MK-HAI [41] consists of 14 items and is a self-report inventory assessing tendencies towards health-related worries or fear of illness over the last six months. The items are scored on a five-point scale from “strongly disagree” (0) to “strongly agree” (4). The total sum score ranges from 0 to 56. The German version of the MK-HAI is a one-dimensional inventory and had high internal consistency (α = 0.93) in a student sample [41].

#### Dimensional obsessive-compulsive scale - short form (DOCS-SF)

The DOCS-SF [42] is a screening instrument for the diagnosis of OCD that consists of two parts. First, the existence of any of four main symptom clusters (e.g. “Unwanted and unpleasant thoughts about germs and contamination and/or repetitive behaviors or mental rituals to prevent contamination”) is indicated, followed by five items on qualitative parameters (e.g., “About how much time have you spent with unwanted thoughts or repetitive behaviors during the last month?”), which are answered on individual six-point scales (e.g., “none at all” (0) to “constantly” (5)). Altogether, these provide a severity rating for all obsessions combined, with a response range from 0 to 25. The mean value for the DOCS-SF in the German-speaking university student sample was 5.55 (*SD* = 5.21) [42] and the scale showed a single-factor structure. In a sample of students, the German version of the DOCS-SF showed good internal consistency (α = 0.89) [42].

#### Eating disorder examination–questionnaire (EDE-Q8)

The EDE-Q8 [43] is a brief 8-item measure covering four domains of eating disorder pathology: Restraint, Eating Concern, Weight Concern, and Shape Concern (two items per subscale) over a four-week period. Items are answered on a seven-point scale from 0 to 6 with different response labels (e.g., describing frequencies, “not a single day” (0) to “every day” (6), or intensities, “not at all” (0) to “considerably” (6)). The total score is calculated as a mean score ranging from 0 to 6. The EDE-Q8 questionnaire showed a high internal consistency (α = 0.93) in a representative German general sample [43].

#### Scale for assessing disgust sensitivity (SADS)

The SADS [44] includes seven items (e.g., “The thought of a disgusting feeling makes me nervous.”) assessing dispositional disgust sensitivity with a 5-point scale from (1) “never true” to (4) “always true”. A total mean score can be calculated, ranging from 1 to 4. The mean value for the SADS in a German-speaking general sample was 1.83 [44]. The unidimensional scale has shown high internal consistency (α = 0.85) in a community sample [44].

#### Somatosensory amplification scale (SSAS)

The SSAS [45]consists of 10 items measuring the attentional focus on bodily symptoms and their interpretation as disturbing (e.g., “Even something minor, like an insect bite or a splinter, really bothers me”), which are rated on a 5-point scale from “not at all” (1) to “extremely” (5). The total sum scores ranges from 10 to 50 and higher scores indicate a stronger somatosensory amplification. In a mixed sample of hypochondriasis outpatients and controls, the SSAS has shown a good internal consistency (α = 0.82) [45].

#### Brief symptom inventory (BSI)

The BSI [46] was administered in the clinical sample and is a short form of the Symptom-Checklist (SCL-90-R) [47] measuring overall and domain-specific psychological distress in 53 items. Items are answered on a five-point scale from “not at all” (0) to “very high” (4). The BSI includes nine subscales: Somatization, Obsession-Compulsion, Interpersonal Sensitivity, Depression, Anxiety, Hostility, Phobic anxiety, Paranoid ideation, and Psychoticism; and three global indices of distress: General Severity Index, Positive Symptom Distress Index, and Positive Symptom Total. Internal consistencies of the subscales ranged from α = 0.71 (Psychoticism) to α = 0.85 (Depression) in a sample of US-American psychiatric outpatients [48].

### Statistical analyses

Analyses were conducted with R (v. 4.1) and RStudio (v. 2023.12.1). Confirmatory factor analyses (CFA) were carried out in both samples to investigate the one-factor and the two-factor structure of the German version of the SPOVI using the *lavaan* package. Models included only main loadings and correlated factors. Diagonally weighted least squares estimation was used to treat the response options as an ordinal scale [49]. The model fit was determined using the Root Mean Square Error of Approximation (RMSEA), Comparative Fit Index (CFI), Tucker-Lewis Index (TLI), and Standard Root Mean Square Residual (SRMR). Following established conventions [50], RMSEA values close to 0.06, CFI and TLI values greater than 0.95, and SRMR values less than 0.08 were regarded as indicators of good model fit. χ² difference tests were conducted to determine whether the two-factor model provided a significantly better fit than the more parsimonious one-factor model. To this end, we also examined the latent factor correlations of the two-factor model, given that a previous validation study indicated insufficient independence of the constructs by very high intercorrelations [29]. Furthermore, Haberman analyses [51] were carried out using the *subscore* package to determine the added value of sub-scores over the SPOVI total score, as indicated by the respective proportional reduction in mean squared error (PRMSE). If values based on the sub-scores (PRMSE_s_) are larger than those based on the total score (PRMSE_x_), they provide a stronger predictor of an individual’s true score. Conversely, if PRMSEs > PRMSE_x_ the sub-scores provide no added value over reporting the total score. Internal consistency was determined with McDonald‘s ω and Spearman-Brown coefficients for the GAD-2 and PHQ-2 [52] using the *psych* package. Correlations of the SPOVI with phobic anxiety (EmetQ-13, BSI phobia subscale^1^), other forms of anxiety (GAD-2, MKHAI, MiniSPIN, BSI anxiety subscale^1^), obsessive-compulsive symptoms (DOCS-SF, BSI subscale compulsiveness^1^), disgust sensitivity (SADS) as well as somatosensory amplification (SSAS, BSI subscale somatization^1^) were investigated as indicators of convergent validity. Correlations with depression (PHQ-2, BDI-II^1^), eating disorder (EDE-Q8), as well as paranoid ideation and psychoticism (BSI subscales^1^), were investigated as indicators of divergent validity. Since psychological data, particularly in psychopathology research, are typically non-normally distributed and robust statistical procedures are recommended [53], we calculated bivariate correlations using the percentage bend correlation coefficient (*r*_pb_), which provides more reliable and robust assessments of the true relationship between variables in case of distributional distortions or statistical outliers [54]. We used the *WRS2* package to calculate percentage bend correlation coefficients, the size of which was interpreted according to Cohen [55]. To find out whether zero SPOVI total scores affected results, we repeated CFA, correlation, and reliability estimation without these participants. Outpatients with specific phobia diagnoses were compared to other diagnoses with the non-parametric Brunner-Munzel test [56] using the *brunnermunzel* package. Generally, effects were considered significant when *p* <.005, as has been recommended by Benjamin et al. [57].

## Results

### Participant characteristics

Out of 580 participants of the mixed community sample, who started the online survey, we excluded 113 (19.5%) datasets with missing values, 11 (1.9%) with an RSI > 2, and 15 (2.6%) with wrong answers on the instructed-response item. Thus, 441 participants entered analyses. In the outpatient sample, out of 622 patients who were invited to fill out the routine diagnostic, 593 patients returned the assessment (95.4%). One hundred twenty-eight (21.6%) participants with missing values in the questionnaires and relevant demographics were excluded. This resulted in a sample of 465 who were analyzed.

Table 1 describes the participant characteristics of both samples. The majority of participants were female (67.7–89.1%), most participants indicated a high level of education (68.4–82.5%), and the average age was 26.63 (mixed community sample) and 34.01 (outpatient sample), with an overall range of 17–79 years. In the outpatient sample (*n* = 274 with available diagnostic data), the most frequent diagnoses were depressive disorders (62.3%), anxiety disorders (30%), and personality disorders (13.4%). Seventeen patients (6.9%) were diagnosed with a specific phobia, see Supplement B for more details, including SPOVI mean scores per diagnostic category.

**Table 1:** Participant characteristics

	Mixed community sample *N* = 441	Outpatient sample *N* = 465
Age, *M* (*SD*), range	26.63 (7.91), 18–79	34.01 (13.43), 17–78
Gender, *n* (%)		
Female	393 (89.1)	315 (67.7)
Male	45 (10.2)	138 (29.7)
Diverse	3 (0.7)	6 (1.3)
No information	–	6 (1.3)
Education^a^, *n* (%)		
Low	11 (2.5)	38 (8.2)
Medium	62 (14.1)	76 (16.3)
High	364 (82.5)	318 (68.4)
Other	4 (0.9)	30 (6.4)
Relationship status, *n* (%)		
Single/divorced/widowed	137 (31.1)	346 (74.4)
In relationship/married	304 (68.9)	119 (25.6)
Occupation, *n* (%)		
Vocational training/school	8 (1.8)	14 (3)
Student	293 (66.5)	88 (19)
Currently working	111 (2.2)	54 (11.7)
Not working	11 (2.5)	279 (60.3)
Other	18 (4.1)	28 (6.1)
No information	–	2 (0.4)
Body mass index (kg/m²), *M* (*SD*), range	22.64 (4.31) 14.2–43.25	25.07 (6.04) 14.7–55.16

^a^ High = A-level and above (tertiary entrance requirements); Medium = secondary school certificate/completed vocational training; Low = less than secondary school certificate/no graduation

### Confirmatory factor analysis (CFA)

The 2-factor model had a good model fit for most indices in the mixed community sample and an excellent model fit in the outpatient sample (see Table 2). All factor loadings were significant, ranging from 0.753 to 0.949 (mixed community sample) and 0.686 to 0.971 (outpatients), see Supplementary Table C1. The one-factor model also demonstrated a good model fit in the mixed community sample and an excellent model fit in the outpatient sample (see Table 2). Factor loadings for the one-factor model were significant, ranging from 0.749 to 0.941 (mixed community sample) and 0.685 to 0.970 (outpatient sample), see Supplementary Table C2.

**Table 2:** Fit statistics for confirmatory factor analysis models

Model	Sample	χ²(df), *p*	RMSEA [95% CI]	TLI	CFI	SRMR
1-factor model	Community sample	231.215 (77), *p* <.001	0.067 [0.058, 0.078]	0.998	0.999	0.044
	Outpatient sample	104.535 (77), *p* =.020	0.028 [0.012, 0.041]	0.999	1.000	0.041
2-factor model	Community sample	204.227 (76), *p* <.001	0.062 [0.052, 0.072]	0.999	0.999	0.041
	Outpatient sample	104.205 (76), *p* =.018	0.029 [0.013, 0.042]	0.999	1.000	0.040

Note: RMSEA = Root Mean Square Error of Approximation, TLI = Tucker-Lewis Index, CFI = Comparative Fit Index, SRMR = Standard Root Mean Square Residual

To determine which of the factor models fit the data better, we first conducted χ² difference tests in both samples. The χ² difference resulted in a significant model difference in favor of the two-factorial model in the mixed community sample (χ²_diff_(2) = 26.988, *p* <.001), whereas the test statistic in the outpatient sample was non-significant (χ²_diff_(2) = 0.33, *p* =.566). Next, we inspected factor correlations in the two-factor models and found that these were almost perfectly correlated in both samples (mixed community sample: *r* =.97, outpatient sample: *r* =.99, *ps* < 0.001). To compare the relative reliability and utility of the subscales to the total score, Haberman analyses were conducted. Results in the outpatient sample showed a higher PRMSE_s_ value for the total score than for both sub-scores (PRMSE_x_). In the mixed community sample, the PRMSE_s_ value for the total score was higher than the “threat monitoring” (F2) sub score but not higher than the “avoidance” (F1) sub score. Details can be found in Supplement C3– C4. Together, these findings indicate that the subscales of the two-factor model do not adequately improve the prediction of true subscale scores compared to using only the total scale score, as in the one-factor model.

CFAs for participants with non-zero SPOVI total scores yielded comparable CFI and TLI values, while RMSEA values were slightly higher, see Supplementary Table C5.

### Reliability and validity

Reliabilities of the SPOVI total scores (mixed community sample: ω = 0.97, outpatients: ω = 0.95) and subscales were high, see Supplementary Table D1. Means, standard deviations, and item-total correlations of SPOVI items for both samples can be found in Supplementary Tables D2– D3.

Table 3 shows the correlation of the SPOVI total score with other relevant constructs. Convergent validity of the SPOVI was supported by large significant correlations with measures of emetophobia (EmetQ-13, *r* =.81), general anxiety (GAD-2, *r* =.54) or illness anxiety (mkHAI, *r* =.59), and disgust sensitivity (SADS, *r* =.75) in the mixed community sample. Contrary to our hypotheses, associations with somatosensory amplification (*r* =.20) and social phobia symptoms (*r* =.22) were relatively smaller. In the outpatient sample, moderate correlations were observed with the phobic anxiety (*r* =.31), anxiety (*r* =.29), and somatization (*r* =.37) subscales of the BSI, which was in support of convergent validity.

**Table 3:** Correlations for specific phobia of vomiting inventory (SPOVI) total score and other measures,* descriptive statistics*,* and internal consistency*

Sample	Measure	Correlation *r*_pb_	M (SD)	Internal consistency (ω, ρ)
Mixed community sample (*N* = 441)	Emetophobia Questionnaire–13	0.81***	33.27 (13.56)	0.94
	Generalized Anxiety Disorder–2	0.54***	2.31 (1.89)	0.85
	Modified Short Version of the Health Anxiety Inventory	0.59***	20.80 (13.57)	0.95
	Mini– Social Phobia Inventory	0.22***	5.20 (3.38)	0.80
	Patient Health Questionnaire–2	0.38***	1.78 (1.66)	0.87
	Eating Disorder Examination–Questionnaire 8	0.15**	1.79 (1.54)	0.92
	Scale for assessing disgust sensitivity	0.75***	2.10 (1.17)	0.95
	Somatosensory Amplification Scale	0.20***	29.51 (7.04)	0.79
	Dimensional Obsessive–Compulsive Scale–Short Form^a^	0.60***	10.76 (6.35)	0.90
Outpatient sample (*N* = 463)	Beck Depression Inventory-Revised	0.22***	22.06 (11.5)	0.92
	Brief Symptom Inventory – Somatization	0.37***	5.66 (5.12)	0.78
	Brief Symptom Inventory – Obsession-Compulsion	0.21***	9.58 (5.84)	0.83
	Brief Symptom Inventory – Interpersonal Sensitivity	0.20***	6.20 (4.06)	0.78
	Brief Symptom Inventory – Depression	0.19***	6.20 (4.06)	0.85
	Brief Symptom Inventory – Anxiety	0.29***	7.33 (5.15)	0.80
	Brief Symptom Inventory – Hostility	0.19***	5.10 (3.82)	0.74
	Brief Symptom Inventory – Phobic anxiety	0.31***	3.86 (4.02)	0.78
	Brief Symptom Inventory – Paranoid ideation	0.20***	4.87 (4.46)	0.77
	Brief Symptom Inventory – Psychoticism	0.26***	5.04 (4.11)	0.73

*Note.* ***p <.001. ^a^*n* = 242 (participants who indicated any obsessive-compulsive thoughts/behaviors in part 1 of the questionnaire)

*r*_pb_ = percentage bend correlation, ω = McDonald’s Omega, ρ = Spearman-Brown coefficient (PHQ-2, GAD-2)

In both samples, divergent validity for the SPOVI was established by relatively smaller correlations with eating disorder symptoms (EDE-Q8, mixed community sample), or paranoid symptoms (BSI subscale paranoid thinking, outpatient sample). SPOVI scores were weakly to moderately correlated with symptoms of depression (PHQ-2, BSI subscale depression) in both clinical and non-clinical samples. Inconsistent results were found for OCD symptomatology, which was largely correlated with the SPOVI in the mixed community sample (*r* =.60), but demonstrated a small correlation in the clinical sample (*r* =.21). The correlation with the BSI subscale psychoticism (*r* =.26) was in a similar range than for anxiety symptoms and relatively lower for paranoid ideation subscale (*r* =.20). The SPOVI did not correlate significantly with body mass index (BMI) in the mixed community sample (*r* =.07, *p* =.124) but significantly negatively in the outpatient sample, albeit with a weak effect (*r* =–.14, *p* =.004).

Correlations for participants with non-zero SPOVI total scores were generally lower across samples, yet the pattern of results was comparable to the total sample, see Supplement E.

### Frequencies and clinical elevations

Across studies, responses in the SPOVI total score ranged from 0 to 56, covering the full range of the scale. Average scores for the total and sub-scales were higher in the mixed community sample than in the outpatient sample, see Supplementary Table D1. Eighty-four (19.1%) respondents from the mixed community sample and almost half of the outpatients (*n* = 207, 44.5%) did not indicate any symptoms of emetophobia (i.e., total score = 0). In both samples, female participants had significantly higher SPOVI scores than male participants (mixed community sample: *d* = 0.91; outpatients: *d* = 0.29), *ps* <.001. Higher scores were significantly positively associated with age (*r* = 0.25, *p* <.001) in the mixed community sample but significantly negatively in the outpatient sample (*r* = − 0.21, *p* <.001). In outpatients, the highest SPOVI total scores were observed for patients with a specific phobia diagnosis (not further specified by type), *M* = 15.82 (18.09), as compared to those without this diagnosis, $\:\widehat{p}$*= (17.08) = 3.49, *p* =.003 $\:\widehat{p}$”=.747 (see Supplement B for descriptives). One-third (34.2%, *n* = 151) of the participants from the mixed community sample scored beyond the proposed cut-off > 10 and around one in ten outpatients (11.8%, *n* = 55).

## Discussion

As no diagnostic instruments for the assessment of emetophobia are available in German yet, this study aimed to psychometrically validate a German version of the SPOVI in both a non-clinical and a clinical sample. Our findings indicated that the German SPOVI is best represented by a unidimensional measure, as opposed to the two-factor model (i.e., F1: avoidance, F2: threat monitoring) of the original version [12] and a Japanese translation [28]. While both the one- and two-factor models in our study yielded comparable (good) model fit and the internal consistencies of the subscales were high, the distinctness of the subscales remains questionable due to their almost perfect correlation in both samples, which was further supported by results of the Haberman analyses. This is consistent with findings of a validation of the English version of the SPOVI in a non-clinical sample by Maack et al. [29] that indicated a similarly high intercorrelation (*r* =.96) and no increment in relative reliability of the two-factor solution. A comparison with the original study is not possible as the authors did not report factor correlations. From a conceptual standpoint, the evidence for one-dimensionality emphasizes the strong connection between threat monitoring or hypervigilance and avoidance as central mechanisms of cognitive models of anxiety disorders and specific phobias in particular [58, 59].

The SPOVI provided good psychometric properties regarding reliability and construct validity in both the community as well as in the outpatient sample. In line with our hypotheses, we found positive associations of the SPOVI with other measures of anxiety. The strong correlation with the EmetQ-13 (*r* =.81) was notably higher than in the study by Boschen et al. [16], (*rs* = 0.25 [controls] – 0.45 [emetophobic patients]) but comparable to the original validation study by Veale et al. [12] in (*r* =.82 [total sample]). These findings confirm concurrent validity between the two measures, also reflecting the overlap in assessing primarily avoidance behavior. At the same time, the variability in correlations underscores the need for further research to determine the redundancy or distinctiveness of the two available self-reports on fear of vomiting (i.e., SPOVI, EmetQ-13) and to assess their incremental clinical utility, for example in diagnostic accuracy. Moreover, other symptom domains of emetophobia – such as cognitions about the meaning and unacceptability of vomiting, anxious somatization, disgust sensitivity, and also functional impairment – could be addressed in future questionnaire developments. Furthermore, we found relatively stronger associations of the SPOVI with general anxiety and illness anxiety as well as with disgust sensitivity, further supporting convergent validity. This pattern is largely consistent with previous validation studies using the SPOVI [12, 16, 29]. The notable difference in the association of the SPOVI and self-reports on OCD symptoms between the two samples should be interpreted cautiously due to the conceptually different measurement instruments (i.e., BSI, DOCS-SF). Previous studies have more consistently found moderate correlations with fear of vomiting [12, 16]. It seems plausible that fear of vomiting can cause repetitive behaviors (e.g., compulsive washing; reassurance seeking, checking others) that overlap with OCD. In clinical cases (i.e., self-reported or clinically diagnosed emetophobic individuals), the comorbidity rate with OCD are varying, which however, is true for other comorbidities as well (e.g., panic disorder) [19, 60]. Moreover, although some aspects of feared or avoided situations (i.e., being sick/vomiting in public) can be shared characteristics of social phobia and emetophobia, our correlational data suggest that the German SPOVI can sufficiently discriminate between social anxiety and the specific fear of vomiting.

The discriminant validity of the SPOVI was supported by relatively low correlations with other psychopathology measures (e.g., assessing depression, paranoid ideation, and eating disorder symptomatology). For example, SPOVI scores correlated weakly positively with scores on the EDE-Q-8. While persons with emetophobia may restrict their food intake due to fear of vomiting, the EDE-Q-8 includes questions on eating restraint related to weight- and shape concerns. Thus, the weak association between SPOVI and EDE-Q-8 scores might reflect these different motivations to restrict food intake. Moreover, while food restriction related to emetophobic fears has been associated with being underweight [11, 20], our findings did not indicate a clear relationship between higher emetophobic tendencies in the SPOVI and lower BMI. Thus, while there seems to be a subgroup of persons with emetophobia that has a low BMI as a result of restricting food intake [11, 20], our findings do not suggest that there is an overall relationship between emetophobic symptoms and body weight. While our results indicate that emetophobic symptomatology can be differentiated from eating disorder symptoms that are typical for persons with anorexia nervosa or bulimia nervosa, a future avenue would be to examine how emetophobia symptoms as measured by the SPOVI can be differentiated from symptoms of avoidant/restrictive food intake disorder (ARFID) [61]. Specifically, restrictive food intake in persons with ARFID is related to several different reasons, including fear of choking or vomiting. Thus, we would expect that measures assessing ARFID symptomatology more strongly relate to measures assessing emetophobia than measures assessing anorexic and bulimic symptoms do.

Fear of vomiting was less associated with somatosensory amplification than we expected, given the conceptual overlap (monitoring of bodily signals and interpretation as signs of illness). This could point towards a rather narrow, phobic threat monitoring (i.e., gastrointestinal symptoms only) in fear of vomiting, compared to the multi-sensory bodily-related threat monitoring as assessed by the SSAS, which is more closely related to hypochondriasis or somatization [62].

The point prevalence of potentially clinically relevant fear of vomiting, as indicated by SPOVI scores > 10, should be regarded cautiously, since we could not confirm this cut-off using clinical diagnoses and since our samples were not representative. Notably, in the mixed community sample, we not only addressed a general sample but also recruited in Facebook groups dedicated to emetophobia (assuming that individuals are more likely to exhibit symptoms of emetophobia), which could explain the rather high average scores and point prevalence (34.2%), limiting the generalizability to the general population. Around one in ten outpatients scored above the proposed cut-off, which suggests that clinically relevant fear of vomiting is not rare in psychotherapy-seeking adults. Also, this rate is comparable to previous estimates in student samples, which ranged from 8.5–9.1.% using the SPOVI [13, 29].

The present study has both strengths and limitations, that should be considered when interpreting our results. Compared to previous studies using the SPOVI or EmetQ-13, our sample was rather large, yet moderate when compared to another previous validation study [29], which did not include a clinical sample, though. Our study provides first and rather large-scale psychometric evidence on the SPOVI in outpatient routine care. However, we did not assess the diagnostic status of emetophobia, which remains crucial for establishing a clinical cut-off. Furthermore, self-report measures differed between the two samples, limiting their transferability. Since our studies were cross-sectional, neither the retest-reliability nor sensitivity to change through treatment was assessed. Other constructs such as interoception, emotional awareness, or magical thinking could be assessed to gain further insights into the mechanisms of fear of vomiting. To improve diagnostic discrimination, further measures of discriminant validity (e.g., personality traits) could have been included. However, the SPOVI performed very similarly between the samples, demonstrating its utility as a screening instrument.

## Conclusions

The German version of the SPOVI has a unidimensional factor structure with sound psychometric properties and provides practitioners with a practical and concise tool for the assessment of fear of vomiting.

## Electronic supplementary material

Below is the link to the electronic supplementary material.

Supplementary Material 1

## Data Availability

All data, scripts, code, and outputs needed to reproduce the results are provided publicly accessible via https://osf.io/d7pgc/.

## Footnotes

1 Based on correlations in the outpatient sample.
