# Supplementary material for: German version of the specific phobia of vomiting inventory (SPOVI): psychometric properties and correlates in a clinical and non-clinical sample
Source: BMC Psychiatry. 2025 Mar 31;25:305. doi: 10.1186/s12888-025-06744-0 (PMC11956176; doi:10.1186/s12888-025-06744-0)
Supplement: Supplementary file 1 — Supplementary Material 1 [file 12888_2025_6744_MOESM1_ESM.docx]

**Supplement A**

**Inventar zur Spezifischen Phobie vor Erbrechen (SPOVI)**

Bitte kreuzen Sie die Antwort an, die am besten beschreibt, wie sich die Angst vor dem Erbrechen in der letzten Woche, einschließlich heute, auf Sie ausgewirkt hat**.**

|  | | **nie** | **selten** | **manchmal** | **oft** | **(fast) immer** |
| --- | --- | --- | --- | --- | --- | --- |
| 1. | Ich habe mir Sorgen gemacht, dass ich oder andere erbrechen könnte/n. | [0] | [1] | [2] | [3] | [4] |
| 2. | Ich habe andere Erwachsene oder Kinder wegen meiner Angst vor dem Erbrechen gemieden. | [0] | [1] | [2] | [3] | [4] |
| 3. | Ich habe bestimmte Situationen oder Aktivitäten wegen meiner Angst vor dem Erbrechen gemieden. | [0] | [1] | [2] | [3] | [4] |
| 4. | Ich habe versucht, Gründe zu finden, die erklären warum mir übel ist. | [0] | [1] | [2] | [3] | [4] |
| 5. | Ich habe bestimmte Gegenstände gemieden, die andere Personen angefasst haben, wegen meiner Angst vor dem Erbrechen. | [0] | [1] | [2] | [3] | [4] |
| 6. | Ich habe mich darauf konzentriert, ob ich mich krank fühle und erbrechen könnte, statt auf meine Umgebung. | [0] | [1] | [2] | [3] | [4] |
| 7. | Ich habe andere beobachtet, um zu sehen, ob sie vielleicht krank sind und erbrechen könnten. | [0] | [1] | [2] | [3] | [4] |
| 8. | Wenn ich denke, dass ich mich übergeben muss, tue ich etwas, um mich vom Erbrechen abzuhalten. | [0] | [1] | [2] | [3] | [4] |
| 9. | Ich habe versucht, jegliche Gedanken oder Vorstellungen über das Erbrechen zu vermeiden oder zu kontrollieren. | [0] | [1] | [2] | [3] | [4] |
| 10. | Ich habe die Menge oder Art meiner Ernährung oder meines Alkoholkonsums wegen meiner Angst vor dem Erbrechen eingeschränkt. | [0] | [1] | [2] | [3] | [4] |
| 11. | Mir war übel. | [0] | [1] | [2] | [3] | [4] |
| 12. | Ich habe darüber nachgedacht, wie ich mich selbst oder andere vom Erbrechen abhalten kann. | [0] | [1] | [2] | [3] | [4] |
| 13. | Ich habe nach Bestätigung gesucht, dass mir oder anderen nicht schlecht wird und ich oder andere nicht erbrechen müssen. | [0] | [1] | [2] | [3] | [4] |
| 14. | Ich bin aus bestimmten Situationen geflüchtet, weil ich Angst hatte, dass ich oder andere erbrechen könnte/n. | [0] | [1] | [2] | [3] | [4] |

**Supplement B**

**Table B**

*Descriptive statistics of SPOVI total scores for selected diagnostic categories in the outpatient sample (n = 247 with complete data)*

| Diagnostic Category | Frequency | | SPOVI | |
| --- | --- | --- | --- | --- |
|  | *N* | *%* | *M* | *SD* |
| Somatoform Disorders | 28 | 11.3% | 3.18 | 6.97 |
| Depressive Disorders | 154 | 62.3% | 3.17 | 7.55 |
| Bipolar Disorders | 2 | 0.8% | 2 | 0 |
| Anxiety Disorders | 74 | 30% | 3.62 | 7.88 |
| Specific Phobia | 17 | 6.9% | 15.82 | 18.09 |
| Obsessive-Compulsive Disorder | 16 | 6.5% | 5.06 | 8.55 |
| Posttraumatic Stress Disorder | 22 | 8.9% | 4.36 | 4.70 |
| Substance use disorders | 6 | 2.4% | 2.17 | 1.17 |
| Eating disorders | 26 | 10.5% | 7.00 | 11.14 |
| Personality disorders | 33 | 13.4% | 3.88 | 8.54 |
| Sexual function disorders | 2 | 0.8% | 0.50 | 0.71 |

**Supplement C**

**Table C1**

*Factor loadings of the SPOVI items for the two-factor model in the two samples*

|  | Mixed community sample | Outpatient sample |
| --- | --- | --- |
| SPOVI item | Factor loading | Factor loading |
| F1: Avoidance Behavior |  |  |
| 2 | 0.946 | 0.956 |
| 5 | 0.893 | 0.971 |
| 3 | 0.946 | 0.939 |
| 7 | 0.912 | 0.893 |
| 14 | 0.943 | 0.934 |
| 10 | 0.874 | 0.865 |
| 9 | 0.909 | 0.957 |
|  |  |  |
| F2: Threat Monitoring |  |  |
| 11 | 0.753 | 0.921 |
| 8 | 0.858 | 0.868 |
| 4 | 0.85 | 0.938 |
| 6 | 0.921 | 0.889 |
| 1 | 0.944 | 0.686 |
| 12 | 0.923 | 0.926 |
| 13 | 0.949 | 0.952 |

**Table C2**

*Factor loadings of the SPOVI items for the one-factor model in the two samples*

|  | Mixed community sample | Outpatient sample |
| --- | --- | --- |
| SPOVI Item | Factor loading | Factor loading |
| 1 | 0.933 | 0.918 |
| 2 | 0.941 | 0.955 |
| 3 | 0.939 | 0.970 |
| 4 | 0.845 | 0.867 |
| 5 | 0.888 | 0.938 |
| 6 | 0.915 | 0.936 |
| 7 | 0.906 | 0.892 |
| 8 | 0.851 | 0.888 |
| 9 | 0.9 | 0.932 |
| 10 | 0.867 | 0.863 |
| 11 | 0.749 | 0.685 |
| 12 | 0.915 | 0.924 |
| 13 | 0.937 | 0.949 |
| 14 | 0.936 | 0.956 |

**Table C3**

*Results of the Haberman analysis for the SPOVI total and sub-scales, mixed community sample*

|  | Alpha | PRMSE.s | PRMSE.x | Added value s |
| --- | --- | --- | --- | --- |
| F1 (avoidance) | 0.9439583 | 0.9439583 | 0.9425251 | 1 |
| F2 (threat monitoring) | 0.9385000 | 0.9385000 | 0.9444389 | 0 |
| Total scale | 0.9668344 |  |  |  |

**Table C4**

*Results of the Haberman analysis for the SPOVI total and sub-scales, outpatient sample*

|  | Alpha | PRMSE.s | PRMSE.x | Added value s |
| --- | --- | --- | --- | --- |
| F1 (avoidance) | 0.8912332 | 0.8912332 | 0.9273650 | 0 |
| F2 (threat monitoring) | 0.9126618 | 0.9126618 | 0.9181344 | 0 |
| Total scale | 0.9428925 |  |  |  |

**Table C5**

*Fit statistics for the one- and two-factor model of the SPOVI for participants with non-zero SPOVI values in both samples*

| Model | Sample | χ²(df), *p* | RMSEA  [95% CI] | TLI | CFI | SRMR |
| --- | --- | --- | --- | --- | --- | --- |
| 1-factor model | Mixed community  (*n* = 357) | 228.95(77),  *p* < .001 | 0.074  [0.063, 0.086] | 0.997 | 0.998 | 0.051 |
|  | Outpatients  (*n* = 258) | 100.48(77),  *p* = .038 | 0.034  [0.009, 0.052] | 0.999 | 0.999 | 0.050 |
|  |  |  |  |  |  |  |
| 2-factor model | Mixed community (*n* = 357) | 205.27(76),  *p* < .001 | 0.069  [0.058, 0.081] | 0.998 | 0.998 | 0.048 |
|  | Outpatients (*n* = 258) | 100.436 (76), *p* = .032 | 0.035  [0.011, 0.053] | 0.999 | 0.999 | 0.050 |

**Supplement D**

**Table D1**

*Mean, SD, and Cronbach’s Alpha (McDonald’s Omega) for the SPOVI total and subscales*

|  | Mixed community sample | | Outpatient sample | |
| --- | --- | --- | --- | --- |
|  | M (SD) | McDonald’s Omega | M (SD) | McDonald’s Omega |
| Total scale | 11.71 (13.48) | .97 | 4.25 (8.63) | .95 |
| Avoidance scale | 4.89 (7.29) | .95 | 2.91 (4.96) | .90 |
| Threat monitoring scale | 6.82 (7.60) | .95 | 1.34 (3.99) | .93 |

**Table D2**

*Item characteristics of the SPOVI, mixed community sample*

| Item | Mean | SD | Item-Total-  Correlation |
| --- | --- | --- | --- |
| 1 | 0.98 | 1.27 | 0.88 |
| 2 | 0.51 | 1.03 | 0.81 |
| 3 | 0.63 | 1.14 | 0.86 |
| 4 | 1.08 | 1.28 | 0.77 |
| 5 | 0.59 | 1.16 | 0.77 |
| 6 | 0.69 | 1.19 | 0.84 |
| 7 | 0.68 | 1.16 | 0.82 |
| 8 | 1.35 | 1.46 | 0.76 |
| 9 | 0.93 | 1.3 | 0.83 |
| 10 | 0.85 | 1.35 | 0.79 |
| 11 | 1.2 | 1.18 | 0.67 |
| 12 | 0.78 | 1.19 | 0.86 |
| 13 | 0.75 | 1.29 | 0.86 |
| 14 | 0.7 | 1.25 | 0.84 |

**Table D3**

*Item characteristics of the SPOVI, outpatient sample*

| **Item** | **Mean** | **SD** | **Item-Total-**  **Correlation** |
| --- | --- | --- | --- |
| 1 | 0.4 | 0.88 | 0.83 |
| 2 | 0.14 | 0.58 | 0.8 |
| 3 | 0.18 | 0.66 | 0.84 |
| 4 | 0.51 | 1.06 | 0.78 |
| 5 | 0.1 | 0.51 | 0.72 |
| 6 | 0.25 | 0.75 | 0.85 |
| 7 | 0.12 | 0.55 | 0.72 |
| 8 | 0.49 | 1.12 | 0.78 |
| 9 | 0.32 | 0.92 | 0.84 |
| 10 | 0.31 | 0.9 | 0.75 |
| 11 | 0.89 | 1.06 | 0.58 |
| 12 | 0.19 | 0.68 | 0.79 |
| 13 | 0.19 | 0.71 | 0.83 |
| 14 | 0.18 | 0.68 | 0.84 |

**Supplement E**

**Table E**

*Correlations for Specific Phobia of Vomiting Inventory (SPOVI) total scores with other measures for participants with non-zero SPOVI total scores*

| Sample | Measure | *r*_pb_ | *p*-value |
| --- | --- | --- | --- |
| Mixed community sample  (*n* = 357) | Emetophobia Questionnaire–13 | .80 | < .001 |
|  | Generalized Anxiety Disorder–2 | .56 | < .001 |
|  | Modified Short Version of the Health Anxiety Inventory | .58 | < .001 |
|  | Mini – Social Phobia Inventory | .19 | < .001 |
|  | Patient Health Questionnaire–2 | .38 | < .001 |
|  | Eating Disorder Examination–Questionnaire | .11 | .031 |
|  | Scale for assessing disgust sensitivity | .73 | < .001 |
|  | Somatosensory Amplification Scale | .16 | .003 |
|  | Dimensional Obsessive–Compulsive Scale–Short Form^a^ | .58 | < .001 |
|  |  |  |  |
| Outpatient sample  (*n* = 258) | Beck Depression Inventory-Revised | .18 | .004 |
|  | Brief Symptom Inventory – Somatization | .23 | < .001 |
|  | Brief Symptom Inventory – Obsession-Compulsion | .10 | .108 |
|  | Brief Symptom Inventory – Interpersonal Sensitivity | .18 | .004 |
|  | Brief Symptom Inventory – Depression | .18 | .004 |
|  | Brief Symptom Inventory – Anxiety | .23 | < .001 |
|  | Brief Symptom Inventory – Hostility | .22 | <.001 |
|  | Brief Symptom Inventory – Phobic anxiety | .23 | <.001 |
|  | Brief Symptom Inventory – Paranoid ideation | .18 | .004 |
|  | Brief Symptom Inventory – Psychoticism | .19 | .002 |
| *Note.* ^a^ *n* = 218 with complete data. *r*_pb_ = percentage bend correlation | | | |
